# Case Study: Transition to a Vegan Diet in an Elite Male Gaelic Football Player

**DOI:** 10.3390/sports9010006

**Published:** 2021-01-05

**Authors:** Daniel Davey, Shane Malone, Brendan Egan

**Affiliations:** 1 Dublin County Board of the Gaelic Athletic Association, Parnell Park, Dublin 5, Ireland; daniel.davey@leinsterrugby.ie (D.D.); shane.malone@mymail.ittdublin.ie (S.M.); 2 Leinster Rugby, Newstead Building A, University College Dublin, Dublin 4, Ireland; 3 Gaelic Sports Research Centre, Department of Science, Technological University Dublin, Dublin 24, Ireland; 4 School of Health and Human Performance, Dublin City University, Dublin 9, Ireland; 5 Florida Institute for Human and Machine Cognition, Pensacola, FL 32502, USA

**Keywords:** athlete, body composition, field sport, running speed, plant-based

## Abstract

Vegan diets are increasingly of interest to athletes, but require a well-planned approach in order to mitigate the risk of potential adverse effects on nutrient intakes, and consequently performance. This case study reports the process of an elite male Gaelic football player (age 25 years; height, 1.88 m; body mass, 87.8 kg; lean body mass, 73.26 kg; body fat, 11.3%) transitioning from an omnivorous diet to a vegan diet at the beginning of a competitive season. The report encompasses key considerations in the planning and provision of nutrition support in this context, in addition to iterations needed based on challenges presented by the athlete. Analysis of nutrient intake (Nutritics Dietary Analysis Software), body composition (dual X-ray absorptiometry; Lunar iDXA, GE Healthcare), and running performance during match-play (global positioning system-based tracking; STATSports Apex) each indicated that with adequate knowledge and education, and appropriate planning, commitment and iterative feedback, the athlete was able to meet nutrition targets on a vegan diet without compromising key performance indicators compared to the omnivorous diet of the previous season. We anticipate that this case study will assist practitioners to recognize the key considerations to address when working with athletes transitioning to a vegan diet.

## 1. Background

Vegan diet (VD) approaches are being increasingly discussed as a potential approach to improve an individual’s health [1], and potentially athletic performance across the spectrum from endurance- to strength- and power-based sports [2,3,4,5]. Anecdotal reports of high-performing athletes who have adopted such a dietary approach [6] suggest that elite-level athletic performance can be achieved while following a well-planned VD. To date, much of the discussion of vegan diets for athletes has centred on environmental benefits and sustainability [4,5], with limited evidence for augmented performance compared to an omnivorous diet (OD) [4]. However, benefits to performance have been proposed through greater carbohydrate intakes, more diverse nutrient intakes, and reduced inflammation and oxidative stress [2,4], although these mechanisms remain to be empirically tested in athletes. 

The substantial changes from an OD in terms of food choices has the potential in turn to substantially alter macronutrient and micronutrient intakes [1,2]. As such, a poorly planned VD can predispose individuals to nutrient deficiencies that could compromise performance in the long-term [2,3]. Therefore, planning a VD to provide adequate energy, protein, essential fatty acids, and micronutrient intakes is challenging, but essential to maintain optimum health and performance of vegan athletes [2,3]. There have been several recent studies describing effects of VDs in athletes and recreational exercisers [7,8,9,10,11], which have demonstrated equivalency with ODs for outcomes such as quality of life and performance [8,9], lower body mass compared to OD [11], and low prevalence of nutrient deficiencies [7,10]. However, there remains an absence of studies describing the applied practice of transition to a VD in elite athletes. This case study focuses on this challenge in an elite male Gaelic football player transitioning from an OD to a VD at the beginning of the 2019 competitive season.

## 2. Presentation of the Athlete

Gaelic football is a field-based team invasion sport that was officially established on the island of Ireland in 1884 [12]. At an elite, albeit amateur, level (termed “senior intercounty”), matches are 70 min in duration plus additional time for stoppages, and are played between two teams of 15 players (with up to 6 substitutes) on a rectangular grass pitch ~145 m in length and ~90 m in width [12,13]. The time commitment to physical, technical and tactical elements is similar to many professional sports, including Australian Rules football and soccer, and the physiological demands of training and match-play are also largely similar to these sports [12,13,14]. 

The athlete is a 25-year-old male elite Gaelic football player currently playing for a senior intercounty team in Ireland. His team are highly successful and have won the sport’s premier elite competition, the All-Ireland Senior Football Championship, seven times in the past nine years. The athlete has played senior intercounty football from the age of 19 and therefore has been engaged in an elite performance structure for ~6 years. He is a highly decorated and core member of the team, with his talents achieving national recognition, having been previously shortlisted for Young Footballer of the Year and being awarded two annual All Star Awards. The athlete plays predominantly in the Full Forward position and is renowned for his power, speed, work rate, and robust playing style. At the time of transitioning to a VD, his physical characteristics were age, 25 years; height, 1.88 m; body mass, 87.8 kg; lean body mass (LBM), 73.26 kg; and body fat, 11.3%.

The athlete’s primary reason for transitioning to a VD centred on his concerns about environmental and climate volatility issues, and the perception that his transitioning to a VD would reduce his personal impact on the environment.

## 3. Overview of Nutrition Plan/Intervention

The athlete had been consuming an OD up until 31 December 2018, at which point he then transitioned to a VD. The athlete has always had a keen interest in nutrition practices to support his performance and would be considered as having a greater interest in the topic than many of his peer group. He had been considering transitioning to a VD for at least six months prior to making the change. Through largely informal conversations with the team nutritionist, he had been informed that there were potentially adverse health and performance outcomes associated with a poorly planned VD. With awareness of these potential issues, the athlete was committed to the necessary preparation and education (both self- and nutritionist-led reading materials) that would be required to implement a VD for athletic performance. Both parties agreed that if the athlete’s health or performance were apparently compromised, then he would revert to a lacto-vegetarian diet as a first countermeasure.

Upon transition to the VD, the athlete was provided with an indicative meal plan reflecting energy- and macronutrient-appropriate requirements of training days and non-training days. This plan was indicative rather than prescriptive, and was designed to meet requirements broadly indicated by recent general nutrition guidelines for athletic performance [15], and specific guidelines for Gaelic football [16], while taking account of the avoidance of animal-derived foods. Key focus points as a function of the VD approach were the aims (i) to provide adequate protein for the athlete’s stature and needs, and in particular providing complementary protein sources; (ii) to enrich the diet with plant sources of long chain n-3 polyunsaturated fatty acids (LC n-3 PUFAs) including walnuts, flaxseeds, and chia seeds, amongst others; and (iii) to address potential nutrient deficiencies through appropriate dietary supplementation.

In turn, the athlete was asked to track his food intake periodically using the MyFitnessPal app (Under Armour, Inc., Baltimore, MD, USA) in order to ensure he was meeting his energy and macronutrient targets, given the change in available food choices and dietary pattern. This nutrient tracking approach was intended to serve both (i) as a means of education and self-reflection for the athlete, and (ii) for the nutritionist as a means of objectively tracking progress in meeting these targets.

Once per week for the first four weeks, formal one-to-one meetings lasting ~30 min in duration were held with the athlete to review the acceptability and practicality of the new dietary pattern. Additionally, an exchange of phone calls and multimedia messaging of meals and snacks were also used on an informal/needs basis for troubleshooting and exchange of ideas around food choices and preparation.

Even a well-planned VD will require dietary supplementation in order to mitigate risk of deficiencies in LC n-3 PUFAs, iron, iodine, calcium, vitamin B12, and vitamin D [1,3]. Mitigating this risk was therefore achieved, in part, through the daily supplementation of a multivitamin dietary supplement (Accovit Performance, ROS Nutrition, Dublin, Ireland) providing iron (24 mg), iodine (150 μg), calcium (200 mg), vitamin B12 (18 μg), and vitamin D (20 μg). Additionally, the athlete supplemented daily with 3 g creatine monohydrate (ROS Nutrition), given the suggestion that athletes consuming diets absent of major dietary sources of creatine from animal flesh have lower muscle creatine concentrations and are most likely to benefit from creatine supplementation [17]. Given the greater requirements for daily protein intake in athletes compared to the general population, and the lower protein density (i.e., protein per kcal of energy intake) of whole food plant-based protein sources [18], the athlete was recommended a plant-based (pea and brown rice) protein powder supplement (Gold Standard 100% Plant Protein, Optimum Nutrition, Dublin Ireland). Advice was primarily for consumption in a post-training shake (gym and field-based sessions), with discretion allowed for consumption on non-training days in the event that his specific protein targets were not being met.

Plant-based protein sources are typically considered less efficient at stimulating anabolic responses than animal-based protein sources, and therefore are less favoured for consumption during recovery from exercise [18]. The potentially lower anabolic properties of plant-based protein sources may be attributed to the lower leucine and total essential amino acid content, limited content of specific amino acids, e.g., lysine, methionine, and/or lower digestibility or absorption [4,18,19]. However, consumption of a larger quantity of plant-based protein powder per serving may mitigate this attenuated response [18], and the overall protein intake of the diet (g·d^−1^) is likely to be more important than the dietary protein source [19]. Indeed, when examining chronic exercise training adaptations as opposed to acute anabolic responses, the evidence for an anabolic advantage of animal-based protein over plant-based protein for skeletal muscle outcomes is equivocal [19]. 

With the athlete actively providing feedback and reflections, the early observations included that (i) tracking his meals and food intake in MyFitnessPal was time-consuming and challenging for the first two weeks, but he became more efficient and accurate with ongoing commitment to the process; (ii) the volume of food was larger than when eating an OD primarily due to the lower protein density of plant-based protein-rich food sources while attempting to meet the assigned target for daily protein intake; (iii) he found it “easy” to meet his daily carbohydrate intake targets of ~4 to 6 g·kg^−1^ i.e., ~350 to 550 g; and (iv) daily fibre intake increased to more than twice the dietary reference value (DRV) of 25 g [20], which coincided with some initial gastrointestinal issues during high intensity exercise performance. This required an adjustment to his pre-training/match meal, and his fuelling preparation on the day before and day of matches. Specifically, during these time periods his daily protein target was reduced by ~35 g (0.4 g·kg^−1^), in addition to reducing fibrous vegetable intake and his choice of carbohydrate-rich foods being advised to be those lower in fibre, e.g., white rice, egg noodles, baby potatoes, amongst others. Replacing fibre-rich protein sources such as lentils and chickpeas with tofu as the pre-exercise protein source was also effective as an approach, with no further gastrointestinal issues reported thereafter.

The team’s training and matches are supported by meals provided on-site by a dedicated catering service, and when travelling for competition, meals are provided in hotel restaurants. In consultation with the team’s nutritionist, vegan-friendly meals were provided by the catering service in each instance, or as pre- and post-match meals in hotel restaurants as appropriate.

## 4. Outcomes of the Implemented Plan

### 4.1. Nutrient Intakes

While the MyFitnessPal app was used to monitor progress informally, a formal analysis (Nutritics Dietary Analysis Software, Dublin, Ireland) of a food diary for a standard field-based training day (Table 1) was undertaken and is presented (Table 2). Compared to a comparable food diary from the 2018 season and similar daily energy intake, daily carbohydrate intake was higher with the VD compared to the OD (479 vs. 406 g), and daily protein intake was lower with the VD compared to the OD (168 vs. 214 g), but by being 1.87 g·kg^−1^, still remained at the upper end of intake recommended for athletes [15].

Intakes of calcium, iodine, vitamin D and vitamin B12 were on the lower limit of intakes without the inclusion of dietary supplements (Table 2), which was unsurprising given that each of these nutrients are noted as being potential nutrient deficiencies when consuming a VD [1,3]. The athlete chose not to commence supplementation with marine-derived LC n-3 PUFAs, namely eicosapentaenoic acid (EPA) and docosahexaenoic acid (DHA). His intake of LC n-3 PUFA in the form of plant-derived alpha-linolenic acid (ALA) was ~3.5 g·d^−1^, which is above the recommended Adequate Intake (AI) of 0.5% of energy intake being from ALA (~2 g·d^−1^) [20]. However, the negligible intake of EPA and DHA was below the recommended AI of 250 to 500 mg per day of EPA+DHA [20]. Given the intake of ~3.5 g·d^−1^ of ALA by the athlete, and the conversion rate from ALA to EPA+DHA usually stated as 5–8% [21], this intake of LC n-3 PUFA in the form of ALA still places the athlete in the appropriate range. High n-6 PUFA intakes may interfere with the efficiency of this conversion, however, and the prevailing opinion is that an n-6:n-3 ratio of ≤4:1 is required to avoid this interference [21]. In the present analysis, n-6 PUFA intake was ~14.2 g, giving an n-6:n-3 ratio of ~4.1:1. Therefore, considering ALA and n-6 PUFA intakes together, the athlete’s LC n-3 PUFA intake pattern would likely be adequate. However, a blood sample analysis to assess n-3 PUFA index (and micronutrient status) would have been useful as an indicator of nutritional adequacy on a personalised basis, but unfortunately was not feasible in this situation.

### 4.2. Body Composition

**Figure 1:**
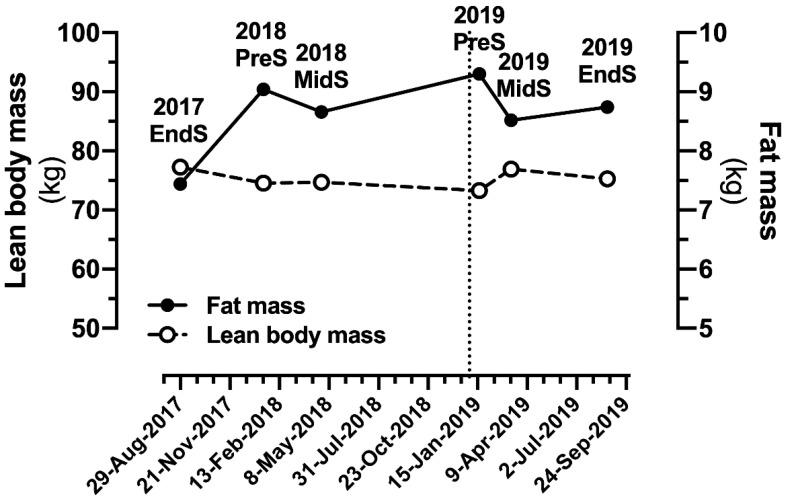
Changes in lean body mass and fat mass in an elite Gaelic games player prior to and after (vertical dotted line) transition to a vegan diet. EndS, end of season; MidS, mid-season; PreS, pre-season.

As part of the team’s regular athlete monitoring processes, body composition was assessed by dual-energy X-ray absorptiometry (DXA; Lunar iDXA, GE Healthcare, Chicago, IL, USA) (Figure 1) throughout the season using standardised preparation, i.e., same time of day within ~1 h of waking, and in a rested and fasted state.

The first DXA scan of the 2019 season was taken on 17th January, ~2.5 weeks after the transition to VD, which also coincided with the end of the off-season period and the beginning of the competitive season, and at a time when the athlete was not participating in a formal strength and conditioning program. Based on the phase of season and previous DXA history, increasing LBM was his primary goal. The athlete was provided specific nutrition targets and a structured muscle hypertrophy program from the Strength and Conditioning coach (CSCS & UKSCA-certified) alongside his field-based training and match commitments. LBM increased by 3.64 kg to 76.90 kg in the following 14 weeks, a value for LBM that he had not reached in the previous competitive season. Over the course of the 2019 season the athlete remained fit to perform in all competitive matches, but in late July he sustained an elbow injury, which limited his ability to complete aspects of the upper-body gym program for 4 weeks and was a likely contributor to his 1.65 kg loss of LBM at the time of late season (August) scan. Percentage body fat was 11.3%, 10.0% and 10.4% at each of the respective scans, which were similar to scans measuring 10.8% and 10.4% in the previous season while consuming an OD.

### 4.3. Assessment of Running Performance during Match-Play

**Figure 2:**
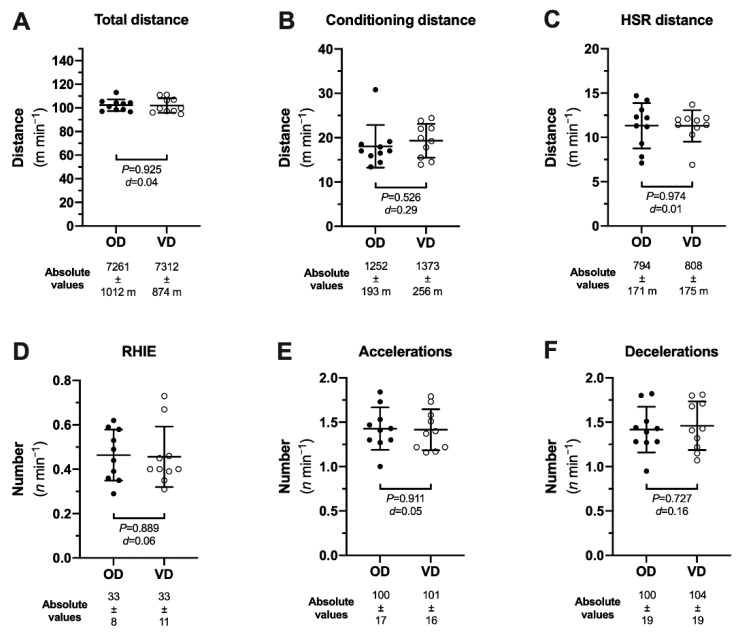
The running performance during match-play in an elite Gaelic games player prior to (OD) and after (VD) transition to a vegan diet. Data presented as mean ± SD. Analysis of distance covered (m) is reported in the following categories; (**A**) total distance; (**B**) conditioning distance (≥4.47 m·s^−1^); (**C**) high-speed running distance (HSR) (≥5.5 m·s^−1^); (**D**) repeated high-intensity efforts (RHIE; defined as three or more high-intensity efforts with less than 21 sec recovery between efforts); (**E**) accelerations (≥3 m·s^−2^); and (**F**) decelerations (≤3 m·s^−2^). Absolute values are reported under each *x*-axis, whereas the figures panels illustrate the data considered with respect of time-on-pitch to provide an intensity (m·min^−1^) measure accounting for potential differences in match duration across the two seasons of match data analysed. Time-on-pitch was 70.8 ± 7.5 min for 2018 OD and 71.5 ± 1.4 min for 2019 VD (*P* = 0.814; *d* = 0.11).

Assessment of running performance during match-play was also used to evaluate if the athlete’s performance was impacted by transition to a VD. The athlete wore a global positioning system (GPS)-tracking unit (STATSports Apex, Firmware: 3.0.10091, Newry, Northern Ireland) sampling at 10-Hz and containing a 400 Hz accelerometer and 10-Hz magnetometer in a total of 20 matches across the 2018 (*n* = 10; OD) and 2019 (*n* = 10; VD) intercounty Gaelic football seasons (Figure 2). Details of the GPS technology use and data analysis have been previously described [22,23,24]. No significant differences were observed when comparing the 2018 and 2019 seasons in any parameter of running performance during match-play (Figure 2).

## 5. Discussion

While there is ample information and research to support potential benefits of a VD and highlight its feasibility when well-planned, even in a sporting context [2,3,4,5], the aim of this case report is to highlight the practical considerations, challenges and performance outcomes of an elite Gaelic football player who transitioned to a VD early in, and throughout, the course of a competitive season. Evidently, with adequate knowledge and education, and appropriate planning, commitment and iterative feedback, the athlete was able to meet the various nutrition targets without compromising key performance indicators in terms of his off-field metrics measured by DXA, or on-field performance, either subjectively reported or assessed using running performance compared to the previous season.

The team nutritionist’s initial reaction when the athlete first stated his intention to transition to a VD was biased towards focussing on the many logistical challenges, potential nutrient inadequacies, and a perceived unlikeliness of success, rather than the potential benefits of consuming a VD as an athlete. Having thoroughly discussed those concerns, the athlete remained adamant and once the nutrition strategy was developed, both parties were united in their approach. Some of the most obvious challenges reported by the athlete were (i) the investment of time in meal planning and food preparation; (ii) the limited vegan-friendly food options in many social situations; (iii) the inability to meet his daily protein targets in a calorie-appropriate manner without the use of a plant-based powdered protein supplement; (iv) the need to be meticulous about his food choices in preparation for intense training sessions and matches. Despite these challenges, within a period of approximately three months, the athlete reported being in a consistent routine, and thereafter found it much easier to meet his daily nutrition targets. Importantly, the athlete’s personal attention to detail, discipline and commitment cannot be underestimated as a major component of the success of his transition to a VD. 

A factor that athletes and practitioners may not always consider is the reaction, and subsequent support or otherwise, from the teammates and management. VDs have historically been viewed with an element of scepticism and phobia due to the unconventional approach to eating [25]. This scepticism is an aspect that must be considered in a team sport environment given the importance of expectations to conform versus encouragement of individual personalities in socialisation processes [26]. A negative reaction by teammates and management has the potential to add stress and self-doubt around the change in diet. Interestingly, for this athlete the response from teammates and management in the initial transition period was both positive and negative, but never malevolent. The athlete is a prominent member of the squad, is highly respected, and is vocal about his commitment to a VD. Once his teammates witnessed his ethical commitment to the diet, and the extent of his efforts invested in his dietary habits, they became more supportive. Some teammates even expressed interest in the potential benefits of a VD, and this led to other members of the squad requesting information on a VD for sport. Ultimately, two other members of the squad subsequently transitioned to a pescatarian diet and a VD, respectively, stating health, environmental and ethical reasons for their change.

Another important factor that facilitated the athlete meeting his nutrition needs was the provision of recovery meals after training and matches. The nutritionist, catering staff and chefs invested significant time creating meal options for the athlete that were vegan-friendly, nutritionally-balanced, and tasty. Additionally, a plant-based powdered protein supplement was used in the formulation of a recovery drink that was prepared by a member of the support team for the athlete to consume immediately after training sessions. The provision of such meals and drinks for recovery are standard practice, albeit non-vegan, for the squad, so the salient point is the recognition of the need for practitioners to be inclusive with the approach to facilitate the needs of such an athlete, despite the additional effort required.

## 6. Conclusions

In summary, despite the many logistical challenges and potential nutrient inadequacies posed by transitioning to a VD, the performance indicators, physiological information and feedback from this athlete’s case study indicated that an elite level of athletic performance and body composition can be maintained after transition to a VD. However, it is also acknowledged that the athlete, in addition to his own motivation and education, had considerable support in terms of personal nutrition consultations, and the support of his teammates and the multidisciplinary support team. Nevertheless, we anticipate that this case study will assist practitioners to recognize the key considerations to address when working with athletes transitioning to a VD.

## Figures and Tables

**Table 1:** Food diary record for field-based training days from omnivorous diet (OD 2018) and vegan diet (VD 2019).

	OD 2018	VD 2019
**Breakfast**	- Large bowl of porridge (100 g oats) topped with blueberries and raspberries and mixed seeds - Slice of sourdough bread and two scrambled eggs	- Smoothie bowl: two bananas, 100 g frozen fruit mix, scoop of vegan plant protein, 1 Tbsp. peanut butter, chia mango pudding and 70 g coconut yogurt
**Snack**	- Two slices of soda bread with 1 Tbsp. peanut butter and medium banana - Fruit yogurt	- Four rice cakes with 2 Tbsp. peanut butter
**Lunch**	- Two baked potatoes with 180 g baked beans, 50 g grated cheese and one large chicken breast	- Large quinoa (200 g) and mixed bean salad with 20 g peanuts, 20 g hazelnuts, lemon juice, fresh herbs, and 1 Tbsp. olive oil - Medium bowl of soup with two large slices of soda bread
**Pre-exercise**	- Four rice cakes with peanut butter, and 1 Tbsp. honey - 400 mL of low fat milk - Handful of dried fruit and mixed nuts (30 g)	- Two toasted pitta breads with 100 g hummus - Two glasses of apple juice
**Recovery**	- Recovery drink: Medium banana blended with 200 mL low fat milk, 2 Tbsp. honey, and one scoop (30 g) of whey protein powder	- Recovery drink: Medium banana blended with 200 mL apple juice, 2 Tbsp. maple syrup, and one scoop (35 g) of plant-based protein powder
**Dinner**	- Two salmon fillets with a large portion of roast vegetables and 200 g white rice	- Vegetable stir-fry with bell peppers, 100 g white rice, 170 g tofu, 100 g edamame beans and 100 g chickpeas

**Table 2:** Energy, macronutrients and selected micronutrients on field-based training days from omnivorous diet (OD 2018) and vegan diet (VD 2019).

	OD 2018 ^a^	VD 2019 ^b^	DRV
**Energy intake**	3632 kcal	3679 kcal	
	41.5 kcal·kg^−1^	41.0 kcal·kg^−1^	
**Carbohydrate**	406 g	479 g	
	4.63 g·kg^−1^	5.34 g·kg^−1^	
**Protein**	214 g	168 g	
	2.44 g·kg^−1^	1.87 g·kg^−1^	
**Fat**	128 g	121 g	
	1.46 g·kg^−1^	1.35 g·kg^−1^	
**Fibre**	45 g	68 g	25 g
**LC n-3 PUFAs**	6.1 g	3.5 g	ALA, 0.5% EI ^c^;
			EPA+DHA, 250 mg ^c^
**Calcium**	1702 mg	732 mg	860 mg ^d^
**Iron**	21 mg	36 mg	6 mg ^d^
**Iodine**	253 μg	28 μg	150 μg ^c^
**Vitamin D**	17.9 μg	9.8 μg	15 μg ^c^
**Vitamin B12**	14.2 μg	0.1 μg	4 μg ^c^

Notes: ^a^ body mass of 87.6 kg at time of recording; ^b^ body mass of 89.7 kg at time of recording; ^c^ as adequate intake (AI); ^d^ as average requirement (AR). DRV, dietary reference value (EFSA, 2017); EI, energy intake.

## Data Availability

The data presented in this case study are available on request from the corresponding author. The data are not publicly available due to personal nature of the data from the subject of the case study.
